# Effect of radioiodine treatment for Graves’ disease on the generation of TSH anti-receptor stimulating antibodies (TSI)

**DOI:** 10.20945/2359-4292-2025-0085

**Published:** 2025-10-28

**Authors:** Lia B. Fiorin, Teresa S. Kasamatsu, Cléber P. Camacho, Gilberto K. Furuzawa, Melissa Furlaneto, Mario Luiz V. Castiglioni, Luiza K. Matsumura, Reinaldo P. Furlanetto, Marcelo C. Batista, Rui M. B. Maciel, Carlos E. S. Ferreira, Carolina C. P. S. Janovsky, João Roberto M. Martins

**Affiliations:** 1 Centro de Doenças da Tireoide e Laboratório de Endocrinologia Molecular e Translacional, Divisão de Endocrinologia, Departamento de Medicina, Escola Paulista de Medicina, Universidade Federal de São Paulo, São Paulo, SP, Brasil; 2 Laboratório de Inovação Molecular e Biotecnologia, Programa de Pós-graduação em Medicina, Universidade Nove de Julho, São Paulo, SP, Brasil; 3 Departamento de Diagnóstico por Imagem, Divisão de Medicina Nuclear, Escola Paulista de Medicina, Universidade Federal de São Paulo, São Paulo, SP, Brasil; 4 Hospital Israelita Albert Einstein, Laboratório Clínico, São Paulo, SP, Brasil; 5 Disciplina de Clínica Médica e Medicina Laboratorial, Departamento de Medicina, Escola Paulista de Medicina, Universidade Federal de São Paulo, São Paulo, SP, Brasil; 6 Centro de Pesquisa Clínica INTEGRA, São Paulo, SP, Brasil

**Keywords:** Graves’ disease, radioiodine, TSH receptor antibodies (TRAb), TSI, Hyperthyroidism, thyroid eye disease

## Abstract

**Objective:**

It is well established that serum levels of TSH receptor antibodies (TRAb) rise after radioiodine (131I) therapy for Graves’ disease (GD). However, it remains unclear whether these post-therapy autoantibodies are predominantly TSH receptorstimulating immunoglobulins (TSI) and how their persistence might affect treatment outcomes.

**Subjects and methods:**

In this prospective study, 39 patients with GD underwent ^131^I therapy. Serum TRAb (measured by competitive electrochemiluminescence, ECLIA) and TSI (measured by an IMMULITE^®^ 2000/2000 XPi TSI assay) were evaluated at baseline and at 1, 2, 3, 6, 9, and 12 months post-therapy. More than 7% increase from baseline was considered a significant rise.

**Results:**

At diagnosis, all 39 patients tested positive for TRAb, while 38 tested positive for TSI. Both TRAb and TSI levels rose significantly between months 2 and 4 post-^131^I, followed by a progressive decline by months 9 to 12. TSI increased in 72% of patients; of these, 93% showed a gradual decrease but remained higher than baseline in 58% at 12 months. Patients with thyroid eye disease (TED), longer disease duration, or higher baseline TSI more frequently exhibited persistent elevation at one year. Despite the persistence of TSI, all patients achieved control of thyrotoxicosis (euthyroid or hypothyroid states).

**Conclusion:**

Radioiodine therapy leads to an increase in TSI, which can remain elevated for up to 12 months in more than half of GD patients. These findings suggest potential benefits of measuring TSI for guiding management decisions, particularly regarding antithyroid drug discontinuation and pregnancy planning.

## INTRODUCTION

Graves’ disease (GD) is an autoimmune thyroid disorder characterized by TSH receptor antibodies (TRAb), which stimulate the TSH receptor and drive hyperthyroidism (^1,2^). Because TRAb are functionally heterogeneous, they may include stimulatory immunoglobulins (TSI), blocking immunoglobulins (TBII), or neutral antibodies (^3-6^). When TSI predominate, they activate adenylate cyclase, causing thyroid hyperfunction (^6,7^).

Radioiodine (^131^I) therapy is a common definitive treatment for GD (^8-11^). Its beta radiation destroys thyroid cells (actinic thyroiditis), triggering a surge in thyroid antigens and often raising levels of autoantibodies such as TRAb (^8,11-13^). TRAb levels typically peak around three months post-treatment and gradually decline, sometimes remaining elevated for five years or longer (^14^). The specific role of TSI within this antibody surge and the implications of persistent TSI levels remain uncertain.

Measurement of TRAb aids in diagnosing GD, predicting relapse of thyrotoxicosis, and guiding therapy (^2,7,15,16^). During pregnancy, assessing TRAb can distinguish GD from gestational hyperthyroidism, guide antithyroid medication choices, and evaluate the risk of fetal or neonatal hyperthyroidism (^15,17^). Conventional TRAb assays (*e.g.*, ECLIA) detect total TSH receptor–binding immunoglobulins, while newer TSI assays measure only stimulatory antibodies (^18^).

Growing evidence indicates that TRAb – even many years after ^131^I therapy – can remain significantly elevated in some patients, including during pregnancy (^19-21^). For instance, very high maternal TRAb levels have been shown to predict neonatal hyperthyroidism or adverse pregnancy outcomes (^21,22^), with one study demonstrating that neonatal thyroid dysfunction occurs in ~5.5% of newborns of mothers who conceived within two years of ^131^I therapy (^23^). Moreover, several case reports highlight that TRAb titers can rise unexpectedly in the late second or even third trimester, sometimes leading to neonatal hyperthyroidism despite stable or low maternal thyroid hormone levels (^21,24^). These observations reinforce the importance of measuring TRAb (and ideally TSI, when available) in women with GD – particularly those with a history of radioiodine therapy – who are planning pregnancy or are already pregnant.

This study evaluated the post-^131^I behavior of both conventional TRAb and TSI in patients with GD, with special attention to whether persistent post-therapy TRAb reflect primarily stimulatory activity. We also discuss the clinical implications of persistent TSI, particularly in women of reproductive age and in patients with thyroid eye disease (TED).

## SUBJECTS AND METHODS

### Study population

We prospectively evaluated 39 consecutive patients (age range 18-70 years) with a clinical and biochemical diagnosis of GD who were referred for ^131^I therapy at our institution. The diagnosis was confirmed by clinical signs/symptoms of hyperthyroidism, diffuse goiter (with or without TED), suppressed TSH, and elevated free T4. TED was assessed using clinical score and NOSPECS and imaging techniques (ultrasound and MRI). None of the patients showed inflammatory activity at the initial presentation, and none of them received corticosteroid prophylaxis before radioiodine therapy. Patients received 10-30 mCi of ^131^I based on disease severity, thyroid size, and comorbidities. Patients received a dose of radioactive iodine due to the failure of medication treatment. All patients were referred to our service at least one and a half years after diagnosis and still in thyrotoxicosis. Those with toxic multi- or uninodular goiters were excluded.

All procedures were performed in accordance with institutional guidelines and the principles of the Declaration of Helsinki. The local Institutional Ethics Committee approved the study, and each patient provided written informed consent.

### Follow-up protocol

Patients were evaluated at baseline (pre-^131^I) and at 1, 2, 3-, 6-, 9-, and 12-months post-therapy. Thyroid volume was measured by the same examiner using ultrasound (Philips Envisor HD, 12 MHz transducer). Levothyroxine was prescribed if TSH exceeded the upper limit of normal or if free T4 fell below normal.

### Laboratory analyses

- **TRAb (ECLIA):** Measured by a competitive electrochemiluminescence assay (Roche Elecsys and Cobas). The positivity cutoff was > 1.75 IU/L.
- **TSI:** IMMULITE^®^ 2000/2000 XPi TSI assay (Siemens), with a positivity cutoff of > 0.55 IU/L.
- **TSH, free T4, total T3:** Measured via electrochemiluminescence or immunofluorimetry (Roche, PerkinElmer), following established reference intervals.
- **Anti-thyroid peroxidase (ATPO), anti-thyroglobulin (ATG):** Measured by Roche Elecsys and Cobas kits. Values were considered positive if > 34 IU/mL for ATPO and > 40 IU/mL for ATG.

For both TRAb and TSI, an increase > 7% from baseline was considered significant.

### Statistical analysis

Continuous variables were evaluated by the Mann-Whitney or ANOVA with Tukey’s post-test, as appropriate. Categorical variables were assessed by chi-square or Fisher’s exact test. Normality was checked using the D’Agostino-Pearson test. Significance was defined as p < 0.05.

### Declaration of generative AI in scientific writing

In order to enhance the clarity and correctness of the language used in this manuscript, we employed ChatGPT version 01 (OpenAI, 2023) for grammar and linguistic revision. This AI-powered tool helped identify typographical errors and improve readability. Nevertheless, the authors take full responsibility for the content and final approval of the submitted version.

## RESULTS

### Baseline Characteristics

We studied 39 patients with an average age of 39.6 years, 76.9% of whom were female, diagnosed with Graves’ Disease on average 3.1 years ago, who received a dose of radioactive iodine due to the failure of medication treatment.

**Table 1** summarizes the baseline clinical and laboratory characteristics. TRAb was positive in all 39 patients (100%) at diagnosis, while TSI was positive in 38/39 (97.4%). 

**Table 1:** Baseline clinical and laboratory characteristics

Parameters	
Age (years-old)	39.6 ± 15.1
Gender (F:M)	30:9
Time of diagnosis (years)	3.1 ± 1.9
Smoking (yes/no)	9/30
Dose of radioiodine (mCi)	16.3 ± 3.6
Presence of TED (yes/no)	33/6
Thyroid volume (mL)	41.6 ± 28.7
TSH (mIU/L)	0.48 ± 1.99
Free T4 (ng/dL)	5.4 ± 1.7
Total T3 (ng/mL)	346.0 ± 234.75
ATG (IU/mL)	218.1 ± 326.2
ATPO (IU/mL)	503.4 ± 194.5
TRAb (IU/L)	14.3 ± 13.1
TSI (IU/L)	24.9 ± 18.2

**Figure 1:**
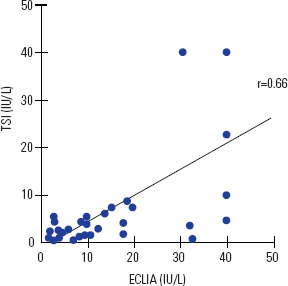
Correlation between the values of TSH anti-receptor antibodies (TRAb) measured by conventional method (ECLIA) and one evaluate only TSH receptor stimulating antibodies (TSI).

**Figure 2:**
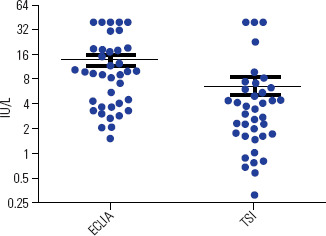
Measurements of TSH receptor antibodies as determined by the conventional method (ECLIA) or evaluated by TSH receptor stimulating antibodies (TSI).

The sensitivity of ECLIA for the diagnosis of GD was 100%, while for TSI it was 97.4%. There was a good correlation between the two methods (r = 0.66) (**Figure 1**). As expected, serum TSI levels were significantly lower than those measured by the conventional method (ECLIA) (**Figure 2**).

### Antibody Behavior After ^131^I

**Figure 3:**
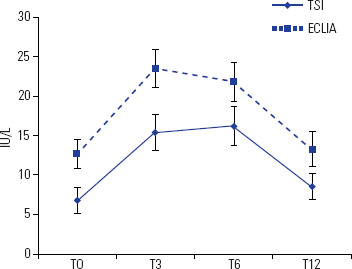
Evolution of TRAb (ECLIA) and TSI levels at different follow-up times

Both TRAb and TSI rose significantly between the second and fourth months (p = 0.0003 and p = 0.001, respectively), followed by a decline toward baseline from months 9 to 12 (**Figure 3**).

TSI levels generally remained elevated slightly longer than TRAb, but both approached pre-treatment values by 12 months (**Figure 3**).

**Figure 4:**
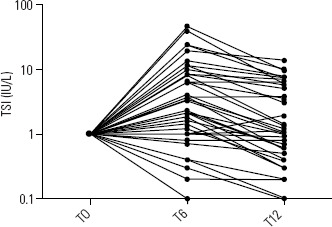
Relative profiles of TSI after treatment with radioiodine.

Among the 39 patients, 28 (72%) showed an initial TSI rise; 93% of these exhibited a subsequent down- ward trend. However, 58% of the 28 still had higher TSI at 12 months compared to baseline (**Figure 4**). 

Patients with TED, longer disease duration, or higher baseline TSI were more likely to display persistent TSI elevation. Importantly, TSI persistence did not prevent control of thyrotoxicosis; over 90% of patients attained euthyroidism or hypothyroidism by one-year post-therapy (**Table 2**).

**Table 2:** Clinical and laboratory characteristics of patients who did and who did not present increasing of TSI after radioiodine

	TSI increased N = 28	TSI did not increase N = 11	p
Gender			
F/M	20/8	10/1	0.4
TED (yes/no)	26/2	7/4	0.04
Smoking (yes/no)	6/22	3/8	0.69
Resolution of hyperthyroidism (yes/no)	24/4	10/1	1.0
Positivity of ATPO (yes/no)	21/7	8/3	1.0
Positivity of ATG (yes/no)	16/12	7/4	1.0
Thyroid volume (mL)	34.5 ± 26.3	28.5 ± 19.8	0.65
Radioiodine (mCi)	16.4 ± 3.8	15.9 ± 3.0	0.85
TSH (mIU/L)	0.09 ± 0.17	1.47 ± 3.67	0.15
Total T3 (ng/mL)	344.7 ± 239.5	349.4 ± 233.4	0.98
Free T4L (ng/dL)	3.1 ± 1.8	3.3 ± 2.2	0.95
TSI (IU/L)	3.2 ± 2.1	13.4 ± 14.8	0.06
Initial TSI (Positive/Negative)	28/0	10/1	1.0
ECLIA (IU/L)	10.8 ± 8.9	21.8 ± 16.8	0.13
Age (years-old)	38.2 ± 15.6	43.3 ± 13.5	0.20
Time of GD (years)	3.4 ± 2.1	2.0 ± 1.0	0.03
ATPO (IU/mL)	296.3 ± 252.4	243.0 ± 228.7	0.52
ATG (IU/mL)	241.8 ± 361.5	99.8 ± 127.8	0.52

TSI: stimulatory thyrotropin-receptor antibodies; M: men; F, female; TED: thyroid eye disease; ATPO: anti-thyroperoxidase antibodies; ATG: anti-thyroglobulin antibodies; ECLIA: anti-thyrotropin.

## DISCUSSION

Our findings confirm that ^131^I treatment induces an increase in TSH receptor antibodies in most patients with GD. Using both a conventional assay (ECLIA, which detects total TRAb) and a stimulatory-specific assay (TSI), we showed that much of the elevated TRAb activity post-^131^I reflects genuine stimulatory immunoglobulins. Despite this persistent elevation of TSI in many patients, nearly all achieved stable thyroid function by the end of follow-up.

These results dovetail with other investigations showing that the rise and persistence of TRAb (especially stimulatory TRAb) can have important clinical implications, particularly for pregnant women or those planning conception (^20,22^). However, not all studies have shown that TSI offers greater predictive value than conventional TRAb assays for certain GD-related complications. In a recent prospective study of 30 patients with newly diagnosed GD, Khamisi et al. likewise found no additional benefit of using TSI instead of TRAb in predicting or managing Graves’ orbitopathy over a 24-month period (^4^). Their data and ours both underscore that while TSI specifically measures stimulating immunoglobulins, the conventional TRAb assay often correlates strongly with TSI and may suffice for routine clinical monitoring.

From a pathophysiologic standpoint, the immune reconstitution or immunologic changes following ^131^I therapy may maintain TRAb production for extended periods, even in clinical remission. Therefore, women with GD treated with radioiodine who wish to become pregnant could be directly impacted (^22^). Our current data – showing frequent TSI elevation at 9-12 months post-^131^I – provide an additional rationale for carefully monitoring women of childbearing age who might become pregnant soon after definitive therapy. This aspect has been recently demonstrated in the study by Priyanka et al. involving 51 women with GD treated with radioiodine. In this study, TRAb levels of > 19.06 IU/L predicted adverse pregnancy outcomes with 100% sensitivity and 93.5% specificity. More importantly, third trimester maternal TRAb levels of > 7.99 IU/L and day three neonatal TRAb levels of > 5.03 IU/L predicted neonatal thyrotoxicosis with 100% sensitivity and 97.4% specificity (^23^).

### Clinical implications

- **Pregnancy Planning:** Because TSI can cross the placenta and potentially cause fetal hyperthyroidism (^19-25^), persistent TSI elevation raises the question of whether a 6-month waiting period after ^131^I is always sufficient before conception (^26^) . Measuring TSI at or near the time of antithyroid drug discontinuation or when counseling women about pregnancy could provide a more individualized risk assessment (^17,27-29^).
- **Thyroid Eye Disease (TED):** Patients with active or preexisting TED may be more susceptible to prolonged rises in TSI, which could exacerbate or increase the risk of worsening ophthalmopathy (^30-32^). Earlier recognition and possible prophylactic measures (*e.g.*, glucocorticoids) may be warranted (^33,34^).
- **Dynamics of TSI vs. TRAb:** TSI tended to remain elevated slightly longer than total TRAb. Although levels eventually declined toward baseline in most patients, 58% showed values above pre-treatment levels at 12 months. This lingering stimulatory autoimmunity underscores the complexity of GD pathophysiology following ^131^I therapy (^4,6,18,35,36^).

### Study limitations

Our main limitation is the relatively small sample size. Larger, prospective studies are needed to confirm these findings, determine optimal timing for TSI measurement, and clarify whether tailoring therapy based on TSI levels reduces fetal hyperthyroidism risk or TED progression.

In conclusion, radioiodine therapy leads to elevated TSI in a significant proportion of patients with GD, with many displaying higher-than-baseline levels one year post-treatment. Although this does not appear to compromise overall treatment success, these data may guide clinical decisions in cases where elevated TSI has critical implications – for instance, in planning pregnancy or managing TED. Our results, together with recent work by others (^23,24,36,37^), highlight the importance of monitoring TSI (or TRAb) for a prolonged period after ^131^I, especially in women of childbearing age.

## Data Availability

datasets related to this article will be available upon request to the corresponding author.
